# Insight into Pathogenic Mechanism Underlying the Hereditary Cataract Caused by βB2-G149V Mutation

**DOI:** 10.3390/biom13050864

**Published:** 2023-05-19

**Authors:** Jing Wu, Silong Chen, Jingjie Xu, Wanyue Xu, Sifan Zheng, Qing Tian, Chenqi Luo, Xiangjun Chen, Xingchao Shentu

**Affiliations:** 1 Department of Ophthalmology, The Second Affiliated Hospital, Zhejiang University School of Medicine, 88 Jiefang Road, Hangzhou 310030, Chinacshelo@zju.edu.cn (S.C.);; 2 Department of Ophthalmology, Zhejiang Provincial People’s Hospital, Affiliated People’s Hospital, Hangzhou Medical College, 158 Shangtang Road, Hangzhou 310053, China; 3 Institute of Translational Medicine, Zhejiang University School of Medicine, 268 Kaixuan Road, Hangzhou 310030, China; 4 GKT School of Medical Education, King’s College London, London SE1 1UL, UK

**Keywords:** congenital cataract, βB2-crystallin, G149V mutation, spectral experiments

## Abstract

Congenital cataracts account for approximately 5–20% of childhood blindness worldwide and 22–30% of childhood blindness in developing countries. Genetic disorders are the primary cause of congenital cataracts. In this work, we investigated the underlying molecular mechanism of G149V point missense mutation in βB2-crystallin, which was first identified in a three-generation Chinese family with two affected members diagnosed with congenital cataracts. Spectroscopic experiments were performed to determine the structural differences between the wild type (WT) and the G149V mutant of βB2-crystallin. The results showed that the G149V mutation significantly changed the secondary and tertiary structure of βB2-crystallin. The polarity of the tryptophan microenvironment and the hydrophobicity of the mutant protein increased. The G149V mutation made the protein structure loose and the interaction between oligomers was reduced, which decreased the stability of the protein. Furthermore, we compared βB2-crystallin WT and the G149V mutant with their biophysical properties under environmental stress. We found that the G149V mutation makes βB2-crystallin more sensitive to environmental stresses (oxidative stress, UV irradiation, and heat shock) and more likely to aggregate and form precipitation. These features might be important to the pathogenesis of βB2-crystallin G149V mutant related to congenital cataracts.

## 1. Introduction

Congenital cataract (CC) refers to a lens opacity that usually exists before, soon after birth, or in childhood [1] and is one of the main causes of blindness in infants and children [2]. Approximately 5–20% of childhood blindness worldwide and 22–30% of childhood blindness in developing countries is attributed to CC [3], accounting for a total of 14 million children according to the World Health Organization [4]. CC is caused by multiple factors, including inherited mutations, intrauterine infection, hypoxia, metabolic disorders, and chromosomal abnormalities [5]. The genetic background accounts for approximately 50% of cases [6]. To our knowledge, the mutation of crystallin genes, cytoskeletal genes, gap junction genes, or transcription factors may lead to CC formation [6,7,8,9]. Untreated CC can lead to sensory deprivation amblyopia. Surgery requires highly trained individuals with unavoidable potential complications and risks, especially in children [10,11]. The timing of surgical treatment is also controversial, producing considerable annual expenditures and an unfavorable prognosis. However, surgical cataract extraction has been the only definitive method of CC treatment. Therefore, further study into the mutations-causing proteins in CC should lead to a greater understanding of the mechanisms implicated in cataractogenesis, providing new strategies for treatment to slow lens opacification.

Crystallins are the most common water-soluble protein in the lens and account for 90% of the lens protein. The crystallin family contains three members, α-, β-, and γ-crystallin and plays a key role in maintaining the transparency and refractive index of the lens [12]. The transmission of light will be blocked when crystallins in the transparent lens are misfolded and precipitation occurs. This can be induced by either intraocular or environmental stresses, such as ultraviolet (UV) irradiation, oxidative, and heat shock. The formation of insoluble protein aggregates in the lenticular tissues and the destruction or structural abnormalities of crystallins lead to the irregular arrangement of lens fiber cells that eventually results in cataract incidence [13,14]. The human lens crystallins represent the most important candidate genes for CC, and various mutations of crystallin have been reported in association with the occurrence of childhood cataract.

The β-crystallin family comprises of seven major lens proteins termed βB1, βB2, βB3, βA1/A3, βA2, and βA4 [15]. β-crystallins are the most common heterogeneous crystallins with a conserved two-domain fold. Each domain contains two Greek-key motifs, which differ in the presence of the C-terminal extension (present in the basic group, none in the acidic group) [16]. β-crystallins form aggregates of different sizes and can self-associate to form dimers or to form heterodimers with other β-crystallins. The CRYBB2 gene is located on the human chromosome 22q11.23 and encodes βB2-crystallin. βB2-crystallin is a late gene and only expressed in the post-natal lens in rodents. In humans, however, βB2-crystallin is expressed at all stages due to its ability to domain swap and subunit exchange with other members of the β-crystallin family to solubilize and stabilize other β-crystallins [17]. There is also a much higher expression level of βB2-crystallin in humans compared to the rodent lens [18,19].

The G149V point missense mutation in βB2-crystallin was first identified in a three-generation Chinese family with two affected members diagnosed with CC [20]. The study discovered that the mutant protein may have a higher hydrophobicity that influences the structure and function of the protein, leading to opacity in the optic lens. Due to the lack of cell culture technology of the lens fiber cells, the detailed mechanism of the protein aggregation pathway in the lens is difficult to elucidate. In this study, we compared βB2-crystallin WT and the G149V mutant with their biophysical properties. We found that the mutation makes βB2-crystallin more sensitive to environmental stresses, including oxidative stress, UV irradiation, and heat shock.

## 2. Materials and Methods

### 2.1. Materials

Taq polymerase, DNA ligase (#6022), and the restriction endonucleases (XhoI (#1094A), NdeI (#1161B), HindIII (#1060A), and BamHI (#1010A)) were from Takara. Sodium dodecyl sulfate (SDS), isopropyl-1-thio-β-D-galactopyranoside (IPTG), bovine serum albumin (BSA), and phenylmethylsulfonyl fluoride (PMSF) were Sigma products. Dulbecco’s Modified Eagle Medium (DMEM) were purchased from Gibco company. DH5α Chemically Competent Cell (#11802ES80) and fetal bovine serum (FBS) (#40130ES76) were from Yeasen (Shanghai, China). The transfection reagent Lipofectamine 2000 (#11668019) and pET-28a (+) plasmid was from Invitrogen. Endofree maxi plasmid kit (#DP117) were obtained from Tiangen Biotech (Beijing, China). The green fluorescence protein (GFP) (#66002-1-Ig) Tag antibody and GAPDH (#60004-1-Ig) antibody were from Proteintech (Wuhan, China). Kanamycin was purchased from Sangon (Shanghai, China). All other reagents were local products of analytical grade.

### 2.2. Plasmid Constructs and Site-Directed Mutagenesis

Human CRYBB2 genes were cloned from the human lens cDNA library as described previously [21,22]. The forward and reverse primers were ccgctcgagatgcctcagatcaccagaccca and cgcggatcccggttggaggggtggaaggcac, respectively. The G149V mutant of CRYBB2 was created by site-directed mutagenesis using overlap extension polymerase chain reaction (PCR) [23,24,25] with forward primers ggtgcagagtgtcacgtgggttggcta and reverse primers tagccaacccacgtgacactctgc. The cloned genes were inserted into the 6xHis-tagged prokaryotic pET-28a (+) vector or the eukaryotic pEGFP-N1 vector for exogenous expression to obtain the recombinant WT and mutated β-crystallins plasmids. The sequences of recombinant plasmids were confirmed by DNA sequencing.

### 2.3. Protein Expression and Purification

The methods of protein expression and purification were as described in previous studies [26,27,28]. The recombinant WT and mutated CRYBB2 proteins were obtained from *E. coli* Rosetta (DE3) which was incubated in a Luria–Bertani medium containing 1000 ng/mL Kanamycin at 37 °C for 4 h. The overexpression of the recombinant proteins was induced by the addition of 0.01 mM IPTG at 16 °C for 20 h. After incubation, the cells were harvested by centrifugation at 10,000× *g*, washed by PBS, and subsequently resuspended in PBS and sonicated by an ultrasonic ice bath (BioSafer, 650-92, Suzhou, China). The *E. coli* BL21 (DE3) precipitates were decomposed by ultrasound to obtain solution fractions containing recombinant protein. The recombinant protein with His-tag was isolated from the supernatant of *E. coli* lysate by Ni-affinity chromatography. The target protein was obtained after further purification by size-exclusion chromatography (SEC) using an ÄKTA Explorer purification system equipped with a Hiload 16/600 Superdex 200 prep-grade column (Cytiva, Marlborough, MA, USA). The purity of the proteins was over 98% by SDS-PAGE and SEC. All protein samples were dissolved in sodium phosphate buffer (SEC buffer: 20 mM Na2HPO4, 150 mM NaCl, 1 mM EDTA, pH = 7.4), and stored at −80 °C. 

### 2.4. Spectral Experiments

Fluorescence spectra were measured by a Hitachi F-4600 fluorescence spectrum fluorometer (Hitachi Co., Ltd., Tokyo, Japan) using a 10 mm path-length corvette with a 5 nm slit width of excitation and emission. Before the test, the cuvette was carefully cleaned, the protein dissolution buffer was used as the baseline control for zero calibration, then 200 μL protein samples were added to the cleaned cuvette for scanning detection. The changes of tryptophan fluorescence peak height and peak position were observed and analyzed. The ANS fluorescence was excited at 380 nm light with the scanning wavelength ranging from 400 to 700 nm. The ANS stock solution with 50 mM concentration was proportionally mixed with the purified protein to a final mole ratio of 75:1 (ANS:protein). The samples were incubated in the dark at room temperature for 30 min before the measurement. Bis-ANS (20 μM) was added to βB2-crystallin (10 μM). The samples were thoroughly mixed and incubated for 10 min before measurements were obtained. Fluorescence emission spectra were recorded at 400–700 nm using an excitation wavelength of 390 nm. The excitation and emission slits were set at 5 nm [29,30,31].

### 2.5. Cell Culture and Transfection

The HeLa cell line was obtained from the American Type Culture Collection (ATCC, Manassas, VA, USA), and HLE cells (SRA 01–04) were obtained from the RIKEN Cell Bank (RCB1591). CRYBB2 and the mutation gene G149V were cloned into the eukaryotic expression vector pEGFP-N1. Cells were cultured in Dulbecco’s Modified Eagle Medium (DMEM, Gibco, Grand Island, NE, USA) basic medium with 10% FBS at 37 °C in a 5% CO_2_ (*v*/*v*) incubator for 24 h. After cultivation to 90–95% confluency, cells were transiently transfected with recombinant plasmids containing either CRYBB2 or the mutation G149V genes using Lipofectamine 2000 (Invitrogen, Waltham, MA, USA) for 4–6 h according to the manufacturer’s protocol. The HeLa cells were then transferred from the serum-free DMEM to fresh DMEM containing an additional 10% FBS and incubated at 37 °C for 24 h.

### 2.6. Immunofluorescence Staining

Details regarding immunofluorescence experiments were performed the same as those described in previously studies [21,32]. A total of 24 h after transfection, the cells were washed by PBS buffer three times for 5 min each time, fixed by 4% paraformaldehyde for 30 min and washed three times again by PBS buffer. Cells were treated by 0.4% Triton X-100 (Sangon Biotech, Shanghai, China) for 15 min and blocked by 10% FBS for 1 h. Immunostaining was performed by rabbit monoclonal anti-p62 antibody (1:200, Proteintech, Wuhan, China) in PBS containing 10% FBS and incubated at 4 °C overnight, followed by staining with Alexa Fluor Plus 555 goat anti-rabbit IgG (1:500, Invitrogen, Waltham, MA, USA) for 2 h at room temperature. The nuclei were stained with DAPI (#36308ES20, Yeasen, Shanghai, China). The exogenously expressed recombinant proteins were detected by the tagged GFP. All immunostaining images and the distribution of these proteins in the HeLa cells were visualized and captured by a Leica DMi8 (Leica, Wetzler, Germany) confocal laser microscope. The percentage of cells with aggregates (cells with GFP highlight aggregation) was analyzed from at least 200 positively transfected cells in 10 randomly selected viewing fields using ImageJ (Collins 2007) and presented as average ± SD.

### 2.7. Western Blot and SDS-PAGE

A 1% NP40 buffer was used to lyse the cells and extract proteins (Sangon Biotech, Shanghai, China). Proteins in the supernatant or precipitate and their total proteins extracted from the transfected cells were run on a western blot. Western blots were quantified with the Image Lab Software (Version 6.0, Bio-Rad Laboratories, Inc., Hercules, CA, USA). A 12% separating gel of SDS-PAGE was used on the purified proteins from *E. coli.* Coomassie blue staining (Coomassie Blue Super Fast Staining Solution, Beyotime, Shanghai, China) was used to determine the concentration of purified protein. For the western blots, (1:1000): anti-GFP (#66002-1-Ig, Proteintech, Wuhan, China) and anti-CRYBB2 (#MB6005, Bioworld, Nanjing, China) antibodies were used.

### 2.8. Molecular Dynamic (MD) Simulations

Molecular dynamics (MD) simulations were performed as described in previous studies [27,28]. In this study, the crystal structure of the human βB2-crystallin (PDB ID: 1YTQ) was used as the template structure to create the starting structure of the WT and G149V mutant. The starting structures of the WT and G149V mutant were created by the molecular simulation program SWISS-MODEL (https://swissmodel.expasy.org/, accessed on 17 September 2022), the simulations were performed using the MD simulation software GROMACS (Linux Version 2.1) with force field CHARMM32. The PyMOL software (The PyMOL Molecular Graphics System, Version 2.2.3, Schrödinger, LLC§, New York, NY, USA) was used to construct the mutated G149V protein from the human βB2-crystallin template (PBD ID: 1YTQ) [33]. The energy-minimized structures were constructed with GROMACS before MD simulations were performed. The MD simulations were conducted in a cubic water box containing 150 mM NaCl with GROMACS. We equilibrated all systems under the condition with a constant number of particles at 310 K for 5 ns in NVT (Canonical ensemble) and NPT (isothermal–isobaric ensemble) conditions. All systems were subsequently run at 2 fs time steps for 100 ns to generate trajectories. The root-mean-square-deviation (RMSD), van der Waals (VDW), electric interaction (ELE), and solvent-accessible surface area (SASA) were analyzed using GROMACS [34]. The PyMOL software was used to display and compare the protein structure after simulation.

## 3. Results

### 3.1. The G149V Mutation Impairs the Structure and Lowers the Solubility of βB2-Crystallin

**Figure 1:**
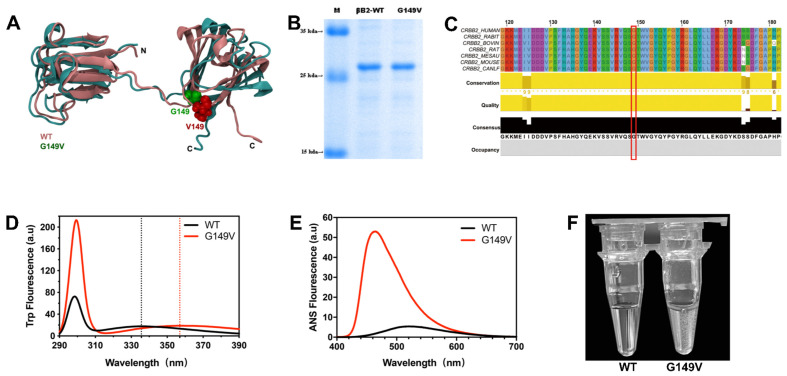
The G149V mutation impairs the structure and lowers the solubility of βB2-crystallin. (**A**) Structural variations between the βB2-WT (dark red) and the simulated G149V (cyan) (PDB ID: 1YTQ). The position of G149 was highlighted by the space-filling model. N and C represent the N- and C-termini of the protein. (**B**) SDS-PAGE analysis showed the purities of the purified protein samples. (**C**) Multiple sequences alignment of CRYBB2 shown for various species. The residues equivalent to G149 in βB2-crystallin were indicated by the red box. (**D**) Intrinsic Trp fluorescence spectra of the βB2-WT and G149V. (**E**) Extrinsic 1-anilino-8-naphthalene sulfonate (ANS) fluorescence spectra of the βB2-WT and G149V. (**F**) The images of protein samples under 4 °C.

To elucidate the aggregation process caused by G149V mutation, MD simulations of the dimeric structures were performed. The dimeric structures of βB2-crystallin are composed of four Greek-key β-sheets divided into two domains. The alignment of the dimeric structures of WT and G149V mutants indicated that the mutation was looser than WT. G149 was also shown to be close to the linking peptide connected by N- and C-domains, which had a great impact on protein folding and structural stability (Figure 1A). According to the SDS-PAGE (Figure 1B), the purity of proteins was very high, both βB2-WT and the G149V mutation were more than 98% pure. The multiple sequence alignments showed that this amino acid was highly conserved in various species (Figure 1C). To evaluate the effect of the G149V mutation, spectroscopy was applied to detect structural changes. Compared with WT, the Trp fluorescence spectra of mutants had a red shift, which represented that the G149V mutation affected the Trp micro-environments and the structure became loose. Further, we also found that G149V mutants had increased resonance light scattering, indicating aggregation formation (Figure 1D). Compared with WT, the ANS and Bis-ANS fluorescence (Figure S1) of the mutant was dramatically higher, which suggests that the G149V mutant had considerable amounts of exposed hydrophobic residues (Figure 1E). During the experiment, we found that G149V mutant protein was easy to precipitate at 4 °C. Precipitation also became more obvious with the increase of concentration, indicating that G149V mutation decreased the solubility of βB2-crystallin (Figure 1F). The maximum solubility of WT was 142.948 ± 0.697 mg/mL, and the maximum solubility of mutant was 23.441 ± 0.053 mg/mL. The results indirectly showed that the tertiary structure of the G149V mutant was loose and unstable, and the protein was easy to misfold and form aggregation.

### 3.2. The G149V Mutation Promotes βB2 Aggregation in Both Human and E. coli Cells

**Figure 2:**
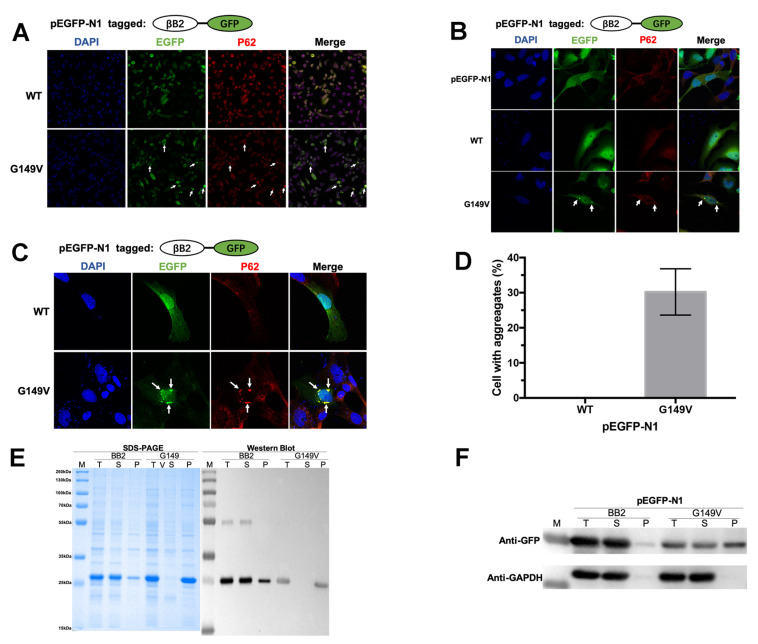
The G149V mutation promoted βB2 aggregation in human HeLa cells and *E. coli* Rosetta cells. (**A**–**C**) Representative confocal laser microscope images with 20× (**A**) and 60× (**B**,**C**) magnification of HeLa cells (**A**,**B**) and HLE cells (**C**) with exogenously expressed WT βB2 and G149V fused by EGFP at the C-terminus. The empty vector of pEGFP-N1 was also transfected and used as the control. The βB2 proteins were visualized by the tagged GFP (green). The aggresomes were recognized by the marker protein p62 (red). Typical aggresomes were indicated by white arrows. The nucleus was stained by DAPI (blue). The white arrows indicated the typical aggregates formed by GFP-G149V, which colocalized with p62. (**D**) Quantitative analysis of protein aggregation in HeLa cells. The percentages were obtained by calculating the ratios of cells with aggregates in ten random viewing fields. The presented data were from three independent experiments. (**E**) SDS-PAGE and western blot analysis of the WT βB2 and G149V overexpressed in *E. coli* Rosetta (DE3) cells. The western blot analysis was performed using antibody against His-tag. (**F**) Western blot analysis of the exogenously expressed WT βB2 and G149V in the HeLa cells. GAPDH was used as the loading control. T: total protein; S: supernatant protein; P: precipitate protein.

The βB2-WT and G149V mutants were exogenously expressed in the human HeLa cell line and *E. coli* Rosetta (DE3) cells to mimic the protein aggregation or precipitation of lens opacity resulting in CC at cell and protein levels (Figure 2). As seen in Figure 2A–C, the exogenously expressed WT βB2 showed the same disperse distribution pattern as the GFP controls, which were both dispersed in the cytoplasm and nucleus of the HeLa cells and the human lens epithelial cells (HLE). For better visualization, tagged GFP (green) was fused to the C-terminus of the WT βB2 protein. The position of the GFP tag does not affect the cellular distribution of βB2. Figure 2 shows that the G149V mutant was prone to form intracellular aggregates in both human and *E. coli* cells, and quantitative analysis showed that the aggregates in G149V mutation were significantly higher than those in WT (Figure 2D). The exogenously expressed G149V mutant of βB2-crystallin forms p62-positive aggresomes in HeLa cells. With confocal microscopy on cells, our results showed that the fused proteins G149V was co-localized with p62, which is an autophagy protein and plays an important role in autophagy and apoptosis. In this study, we found the p62-positive with G149V may indicate that the fused proteins G149V aggregates could be toxic to the cells and may induce cell apoptosis and necrosis.

SDS-PAGE and western blot analysis using antibodies against His indicated that WT βB2 could be successfully obtained from the supernatant fractions of *E. coli* cells (Figure 2E), while the G149V mutated proteins existed in the precipitation fractions, which was consistent with the results from the western blot shown in Figure 2F. Western blot was used to analyze the exogenously expressed protein and was performed with an anti-GFP antibody (Figure 2F). Consistent with the results from confocal microscope images, western blot analysis showed that βB2 protein mainly existed in the supernatant fraction of cell lysates, while the G149V mutated proteins existed in both the supernatant and precipitation fractions.

### 3.3. The G149V Mutation Intracellular Aggregates Were Promoted by H_2_O_2_

**Figure 3:**
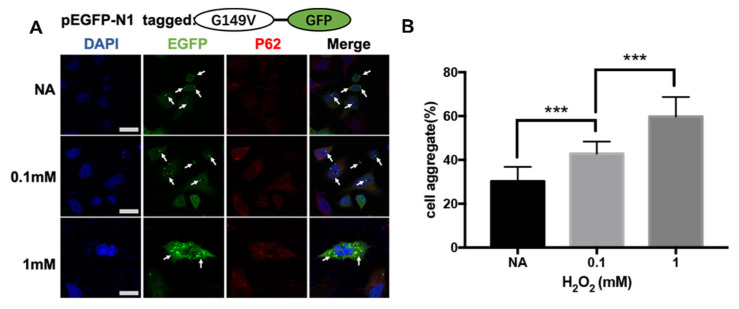
G149V aggregates could be promoted by H_2_O_2_. (**A**) Representative confocal images of cells expressing G149V treated with 0.1 mmol/L or 1 mmol/L hydrogen peroxide. The G149V proteins were visualized by the tagged GFP (green). The aggresomes were recognized by the marker protein p62 (red). The nucleus was stained by DAPI (blue). The white arrows indicate the typical aggregates formed by GFP-G149V. Aggregates in cells increased with increase in hydrogen peroxide concentration. (**B**) Quantitative analysis of the percentage of cells with protein aggregates. Percentage of cells with protein aggregation increased with an increase in hydrogen peroxide concentration. (***, *p* < 0.001).

To study the scientific phenomena of G149V mutants in a cellular environment, the mutant gene was inserted into a eukaryotic expression vector according to the methods described above. The effect of hydrogen peroxide on protein aggregates in HeLa cells containing overexpressed G149V mutant was studied by representative confocal images. As previously mentioned, the distribution pattern of normal WT βB2 crystallin protein in both cytoplasm and the nucleus was similar to that of control cells overexpressing GFP. However, the mutant G149V crystallin protein forms a microscopically visible protein aggregate in cells. In this study, we found that hydrogen peroxide promoted the formation of intracellular aggregates (Figure 3A). Quantitative analysis indicated that the aggregates in the cells increased with the increase of hydrogen peroxide concentration (Figure 3B).

### 3.4. The G149V Mutation Intracellular Aggregates Were Promoted by UV Irradiation

**Figure 4:**
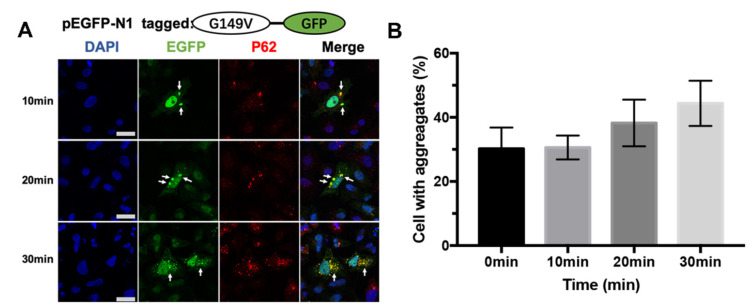
G149V aggregates could be promoted by UV irradiation. (**A**) Representative confocal images of cells expressing G149V treated with 10 min, 20 min, and 30 min UV irradiation. The G149V proteins were visualized by the tagged GFP (green). The aggresomes were recognized by the marker protein p62 (red). The nucleus was stained by DAPI (blue). The white arrows indicated the typical aggregates formed by GFP-G149V. The aggregates in the cells increased with prolonged exposure to UV light. (**B**) Quantitative analysis of the percentage of cells with protein aggregates. The percentage of cells with protein aggregation increased with prolonged exposure to UV light.

The effect of UV on the aggregatory property of βB2 HeLa cells transfected with the mutant G149V protein was evaluated using the UV irradiation model. UV irradiation-induced aggregation was observed by representative confocal images. HeLa cells cultured in the 12-well cell cluster were placed on ice to avoid the effect of temperature variations induced by the light during the UV irradiation exposure. With the prolongation of UV irradiation time, the phenomenon of protein aggregation becomes increasingly obvious. The number of cells with aggregates increased alongside the protein aggregation in a single cell (Figure 4A). The quantitative analysis of the ratio of cells with aggregates indicated that UV irradiation induced a significant increase in the propensity to aggregate (Figure 4B).

### 3.5. The G149V Mutation Intracellular Aggregates Were Promoted by Heat Shock

**Figure 5:**
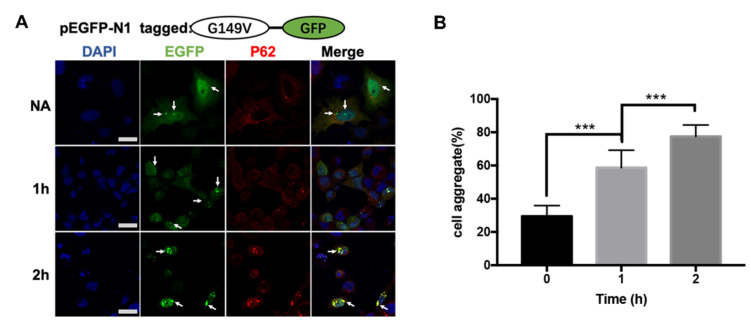
G149V aggregates could be promoted by heat shock. (**A**) Representative confocal images of cells expressing G149V treated with 1 h and 2 h of heat shock. The G149V proteins were visualized by the tagged GFP (green). The aggresomes were recognized by the marker protein p62 (red). The nucleus was stained by DAPI (blue). The white arrows indicate the typical aggregates formed by GFP-G149V. Aggregates in the cells increased with prolonged exposure to heat shock. (**B**) Quantitative analysis of the percentage of cells with protein aggregates. The percentage of cells with protein aggregation increased with the prolonged exposure to heat shock (***, *p* < 0.001).

The increased propensity to aggregate induced by the heat shock was further confirmed by protein aggregation studies. Consistent with the previous stress treatment, βB2-crystallin was prone to aggregate during heat shock. The G149V mutate protein was sensitive to heat-induced aggregation and the formation of protein aggregates increased over time at high temperatures. The culture dish containing HeLa cells with transfected G149V protein was treated at 42 °C in a continuous heating device. Cells with one hour of heat treatment contained more aggregates than cells treated for two hours (Figure 5A), the quantitative analysis of cell aggregation rate showed that the cell aggregation in G149V mutation induced by heat shock increased significantly with time (Figure 5B).

### 3.6. G149V Mutation Significantly Altered the Stability of βB2-Crystallin

**Figure 6:**
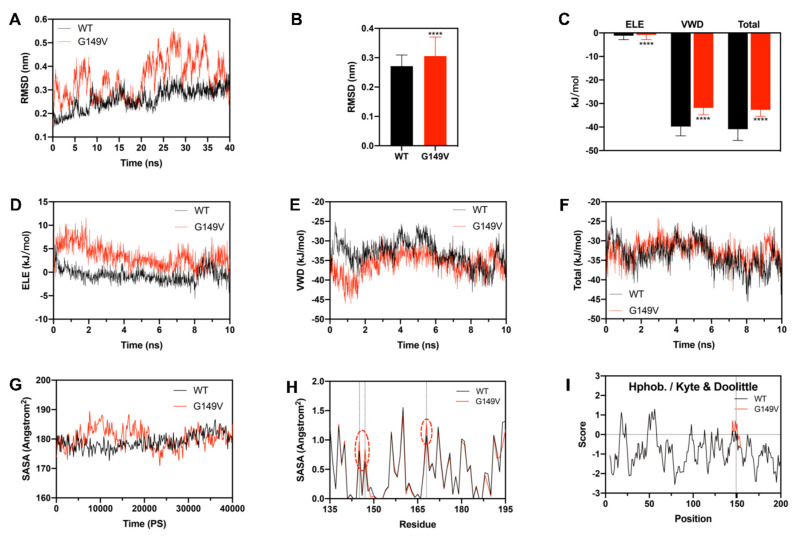
G149V mutation destroyed the stability of βB2-crystallin. (**A**,**B**) Time-course changes of the RMSD values during simulations. (**C**–**F**) Average subunit binding energies calculated from the simulated structure. (**G**,**H**) The time course and residue (residue 135 to 195) SASA of the simulated structure. (**I**) The hydrophobicity (Hphob./Kyte and Doolittle) of the simulation structure (****, *p* < 0.0001).

The G149V mutation was localized in the fourth Greek-key motif, and Greek-key motifs played a role in protein homeostasis [35]. βB2-crystallin exists as oligomers ranging from dimers to octamers in the natural state, of which the most common are dimers and tetramers. The molecular mechanism of the aggregation process caused by the G149V mutation was performed by MD simulation. The RMSD of the G149V mutant changed significantly at the beginning, especially at 20–35 ns (Figure 6A), which was much higher than that of WT βB2 protein (Figure 6A). During the last 100 ns, the average RMSD of G149V was significantly higher than that of the WT (Figure 6B). This indicated that the mutation lowered the structural stability of the protein. It can be seen from Figure 6C that the hydrophobic effect (VDW force) was the main force of βB2-crystallin dimer polymerization to form a tetramer. According to the binding energy curve (Figure 6D–F), although the hydrophobic interaction and electrostatic interaction between the mutant dimers were initially stronger than that of the WT protein, the electrostatic binding energy (Coulomb force, ELE), hydrophobic binding energy, and total binding energy between the two dimers in G149V decreased significantly over the course of the simulation (only the first 10 ns of the simulation time course is shown in the Figure 6D–F). From the SASA, the G149V mutant had higher and more fluctuant SASA compared to the WT, which increased significantly between 10,000 ps and 20,000 ps (Figure 6G). The G149V mutant also had higher residue SASA around G149 and K168 (Figure 6H). The hydrophobicity of amino acids reflects the folding of proteins. According to Figure 6I, the G149V mutant had higher hydrophobicity at nearly 149. These results indicated that the interaction between the dimers of the mutant was destroyed and the stability of the protein was decreased. This may be attributed to the change in binding energy and secondary structure alterations, which was consistent with the fluorescence spectrum results.

## 4. Discussion

βB2-crystallin is the important and high content crystallin in the mammalian lens [36]. In vertebrate lenses, β-crystallins exist as large homomers or heteromers ranging from dimers to octamers [37]. The normal structure and function of the lens proteins, as well as the highly ordered arrangement between them (believed to play an important role in the maintenance of optical properties of the lens throughout the individual’s lifespan), are indispensable for maintaining lens transparency [38]. In this research, we investigated the roles of the G149V mutation of βB2-crystallin in CC. The results showed that the G149V mutation significantly changed the secondary and tertiary structure of βB2-crystallin. The polarity of the tryptophan microenvironment and the surface hydrophobicity of the mutant protein increased. The G149V mutation made the protein structure loose and the interaction between oligomers reduced, which decreased the stability of the protein. Under the stress of thermal, oxidation, and UV irradiation, the protein was more likely to aggregate and form precipitation. Our observations strongly suggest that the accumulation and deposition of crystallin was a common pathological feature of various cataracts.

Previous studies have shown that changing the hydrophobicity of the C-terminal and perturbing the Greek-key motif will lead to the synthesis disturbance of homologous/heterologous oligomers between lens proteins, resulting in different degrees of CC [39]. The G149V mutation in this study was first found in a Chinese family with CC of two generations. The proband was a CC patient with a small cornea, posterior segment defect, and glaucoma. It was reported that the mutant increased the hydrophobicity of lens protein, which was consistent with our research results. The mutation reduced the solubility of the protein, which may affect the structure and function of the protein, leading to lens opacity.

In our research, the G149V mutation led to a significant increase in the exposure of surface exposed, hydrophobic surface area when compared to the WT protein by spectroscopic experiments. The G149V mutation had also been found to have deleterious effects on βB2-crystallin structure, thermal stability, resistance to UV light, and oxidative stress. G149V mutation led to decreased stability against thermal, oxidative, and UV irradiation, which may destroy the formation of a tetramer of βB2-crystallin. In summary, the results of our study demonstrate that the mutations in βB2-crystallin may lead to cataracts by altering the native structure/surface properties, decreasing the stability, promoting aggregation, and/or increasing its sensitivity against the UV irradiation, oxidative stress, and thermal stability.

Both β- and γ-crystallins are defined by four Greek-key motifs divided into two domains [40,41]. The integrity of the conserved Greek-key motifs plays a vital role in the structure, stability, and function of β/γ-crystallins as well as the onset of CCs caused by mutations. A large number of previous studies have shown that the mutation in the fourth Greek-key motif of βB2-crystallin resulted in a significant change in the stability of the protein [28,39]. It has been well established that β-crystallins can form both homomers and heteromers. In this study, we found that the G149V mutation greatly destroyed the interaction between oligomers and promoted the formation of abnormal aggregates. Our results not only provide insight into the molecular mechanism underlying the hereditary cataract caused by the G149V mutation but also highlight the important role in βB2-crystallin oligomerization formation and its stability.

At the protein level, cataracts are closely related to the deposition of soluble structural proteins on the lens. Large aggregates directly scatter light and/or have toxic effects on cells, which disturbs the normal structure and function of the lens. Unlike the thoroughly studied monomeric γ-crystallin mutation, most of the molecular mechanism of crystallin mutation is still unclear, which may be partly due to the complex folding pathway of multimeric proteins and the dynamic balance of diversity between homo/hetero-oligomers. In this study, we found that the G149V mutation affected the secondary and tertiary structure of the protein, loosened the protein structure, destroyed the Greek-key motif, disturbed the interaction between oligomers, increased the sensitivity to stressors including oxidative, UV, and heat shock, and formed abnormal aggregates. In summary, we can infer that the abnormal aggregation of βB2 crystallin oligomers may be an important mechanism of CCs caused by a genetic mutation.

## Data Availability

The data presented in this study are available on request from corresponding authors. The data are not publicly available due to privacy and ethical restriction.

## Supplementary Materials

The following supporting information can be downloaded at: https://www.mdpi.com/article/10.3390/biom13050864/s1, Figure S1: Bis-ANS fluorescence spectra of the βB2-WT and G149V.
