# Supplementary figures and images for: Insight into Pathogenic Mechanism Underlying the Hereditary Cataract Caused by βB2-G149V Mutation
Source: Biomolecules. 2023 May 19;13(5):864. doi: 10.3390/biom13050864 (PMC10216223; doi:10.3390/biom13050864)

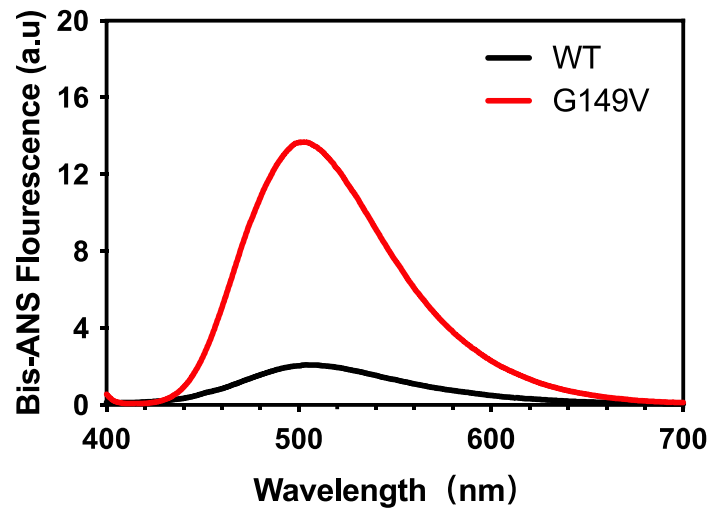

Figure S1. Bis-ANS fluorescence spectra of the  $\beta$ B2-WT and G149V.

Supplement: Supplementary file 1 [file biomolecules-13-00864-s001.zip › biomolecules-2050495-supplementary.pdf]
